# Antimicrobial Susceptibility Pattern of Ceftriaxone-Sulbactam-EDTA Against Multidrug-Resistant Gram-Negative Bacilli From ICU Clinical Isolates

**DOI:** 10.7759/cureus.114898

**Published:** 2026-08-20

**Authors:** Vaishnavi G Chavan, Satyajeet Pawar, Satish Patil

**Affiliations:** 1 Department of Microbiology, Krishna Institute of Medical Sciences, Krishna Vishwa Vidyapeeth (Deemed To Be University), Karad, IND

**Keywords:** antimicrobial resistance, antimicrobial stewardship, ceftriaxone-sulbactam-edta, extended-spectrum beta-lactamase, gram-negative bacilli, intensive care unit, metallo-beta-lactamase, multidrug-resistant

## Abstract

Background

Antibiotic therapy remains the mainstay for the management of bacterial infections. However, the rapid emergence of multidrug-resistant (MDR) organisms has become a major clinical concern, leading to limited treatment options and increased healthcare burden. Ceftriaxone-sulbactam-EDTA (CSE) is a cephalosporin-beta-lactamase inhibitor combination containing disodium edetate, an antibiotic resistance breaker that may help restore antibacterial activity against resistant Gram-negative organisms. This study aimed to evaluate CSE as a potential therapeutic option by assessing its susceptibility pattern against MDR-Gram-negative bacilli (GNB) isolated from intensive care unit patients.

Material and methods

A laboratory-based study was conducted on 92 MDR-GNB isolates from ICU patients at Krishna Charitable & Medical Research Centre, Karad, India. Clinical specimens, including urine, endotracheal tube secretions, sputum, pus/wound swabs, blood, and body fluids, were processed using standard microbiological methods. Bacterial isolates were identified, and antimicrobial susceptibility testing was carried out as per Clinical and Laboratory Standards Institute recommendations. Phenotypic detection of extended-spectrum beta-lactamase (ESBL), metallo-beta-lactamase (MBL), and AmpC beta-lactamase production was performed to characterize resistance mechanisms. Additionally, the susceptibility profile of MDR-GNB isolates to CSE and other commonly used antimicrobial agents was evaluated.

Results

The study analyzed a total of 92 MDR-GNB isolates. The highest number of isolates were observed in the 31-40 and 41-50 age groups, with male predominance. Urine was the most common specimen source, with 39 (42.39%) isolates, followed by endotracheal tube secretions, with 20 (21.74%), and sputum, with 11 (11.96%) isolates. *Pseudomonas aeruginosa* was the predominant organism, followed by *Klebsiella pneumoniae,* *Klebsiella oxytoca, Acinetobacter baumannii, Escherichia coli,* and *Enterobacter cloacae complex. Among the isolates, *51 were ESBL producers, 48 were MBL producers, and 38 were AmpC producers, indicating a significant burden of beta-lactamase-mediated resistance. High resistance was observed against meropenem (94.57%), netilmicin (93.48%), and ciprofloxacin (90.22%). Nitrofurantoin showed good activity among urinary isolates, with 35 (89.74%) susceptible. Co-trimoxazole showed susceptibility in 6 (14.63%) of 41 tested isolates, while tigecycline showed susceptibility in 5 (12.20%) of 41 tested isolates. CSE showed substantial *in vitro* activity, with 80 (86.96%) isolates susceptible and 12 (13.04%) resistant.

Conclusions

MDR-GNBs were frequently isolated from ICU patients, mainly from urine and respiratory specimens, with *P. aeruginosa* and *K. pneumoniae* being the most common organisms. The presence of ESBL, MBL, and AmpC producers highlights the serious burden of antimicrobial resistance. CSE showed promising *in vitro* activity against MDR-GNB isolates and may be considered when guided by susceptibility testing. Strict infection control practices, routine resistance screening, and antimicrobial stewardship are essential in ICU settings.

## Introduction

Multidrug-resistant Gram-negative bacilli (MDR-GNB) are a major global healthcare concern because they are associated with increased morbidity, mortality, prolonged hospital stay, treatment failure, and higher healthcare costs [1]. Globally, bacterial antimicrobial resistance was associated with an estimated 4.95 million deaths in 2019, including 1.27 million deaths directly attributable to antimicrobial resistance [1]. The World Health Organization has identified carbapenem-resistant *Acinetobacter baumannii*, carbapenem-resistant Enterobacterales, and third-generation cephalosporin-resistant Enterobacterales as priority bacterial pathogens, emphasizing the growing threat posed by MDR-Gram-negative infections [2].

In intensive care units (ICUs), MDR-GNB infections are particularly important because critically ill patients are frequently exposed to invasive devices, mechanical ventilation, urinary catheters, central venous lines, prolonged hospitalization, and broad-spectrum antibiotic therapy. Common Gram-negative organisms isolated from ICU patients include *Escherichia coli,* *Klebsiella pneumoniae, Pseudomonas aeruginosa, A. baumannii*, and *Enterobacter cloacae*. According to the standard definition proposed by Magiorakos AP et al., multidrug resistance is defined as acquired non-susceptibility to at least one antimicrobial agent in three or more antimicrobial categories [3].

The development of antimicrobial resistance in GNB is multifactorial. Important mechanisms include production of β-lactamase enzymes, alteration of drug targets, reduced outer membrane permeability due to porin loss, increased efflux pump activity, and biofilm formation. Among these, β-lactamase enzymes play a major role because they hydrolyze β-lactam antibiotics and reduce the efficacy of penicillins, cephalosporins, monobactams, and carbapenems. Extended-spectrum beta-lactamases (ESBLs), AmpC beta-lactamases, metallo-beta-lactamases (MBLs), and carbapenemases have contributed significantly to the rising prevalence of MDR infections in hospital settings [4].

The increasing prevalence of carbapenem-resistant Gram-negative organisms has created an urgent need for alternative therapeutic options that can preserve the efficacy of existing antibiotics while reducing dependence on carbapenems and polymyxins. Ceftriaxone-sulbactam-disodium edetate (CSE-1034) is a novel antibiotic-adjuvant combination developed to overcome multiple resistance mechanisms in MDR-GNB. Ceftriaxone, a third-generation cephalosporin, inhibits bacterial cell wall synthesis, whereas sulbactam acts as a β-lactamase inhibitor that protects ceftriaxone from enzymatic degradation. Disodium edetate (EDTA) functions as a metal-chelating agent that inhibits MBL activity, disrupts biofilm formation, enhances outer membrane permeability, and facilitates increased antibiotic penetration into bacterial cells [5].

Several studies have reported promising *in vitro* activity of the ceftriaxone-sulbactam-EDTA (CSE) combination against MDR-GNB, including ESBL- and MBL-producing organisms [5,6]. In this *in vitro* study, the antimicrobial activity of ceftriaxone-sulbactam-disodium edetate (CSE-1034) was evaluated against MDR-GNB isolated from ICU patients. CSE-1034 is an antibiotic-adjuvant combination approved by the Drug Controller General of India for clinical use in India [7]. The available evidence for CSE is mainly based on limited regional studies, predominantly from India. Therefore, CSE is considered an emerging antimicrobial option, and further large-scale international clinical studies are needed to confirm its efficacy, safety, and role in routine clinical practice. Clinical studies have suggested that CSE may help manage infections caused by MDR-GNB and reduce dependence on carbapenems. However, data on the *in vitro* susceptibility of MDR Gram-negative isolates to CSE remain limited across different healthcare settings. Therefore, the present study evaluated the *in vitro* susceptibility of MDR Gram-negative isolates from ICU patients to CSE in a tertiary care hospital. Although previous Indian studies have reported CSE activity against MDR-GNB, this study provides additional local susceptibility data from our institution, which may support antimicrobial stewardship and guide empirical treatment decisions in similar healthcare settings [8,9].

## Materials and methods

Study design and specimen collection

This laboratory-based investigation was carried out from August 2024 to March 2025 at the Department of Microbiology, Krishna Institute of Medical Sciences and Krishna Charitable & Medical Research Centre, Karad. The study was approved by the Institutional Ethics Committee of Krishna Institute of Medical Sciences, Karad (Protocol No.: KVV/IEC/05/2024). A total of 370 clinical specimens collected from ICU-admitted patients at Krishna Hospital were processed. Of these, 228 specimens showed bacterial growth, including 119 Gram-negative isolates. Based on antimicrobial susceptibility testing, 92 isolates were identified as MDR-GNB and were included in the final analysis, while 27 non-MDR Gram-negative isolates were excluded. Data were collected using a structured proforma to document patient demographic details, clinical information, ICU admission details, specimen type, bacterial isolate, phenotypic resistance profile, and antimicrobial susceptibility pattern. The analysis included all clinical specimens received during the study period, including pus, endotracheal secretions, sputum, urine, blood, and body fluids.

Isolation and antimicrobial susceptibility testing

All clinical specimens received from ICU-admitted patients, including blood, urine, respiratory specimens, wound swabs/pus, and other body fluids, were processed according to standard microbiological procedures appropriate for each specimen type. The specimens were inoculated onto appropriate culture media, including Blood agar and MacConkey agar, and incubated aerobically at 37°C for 18-24 hours. After incubation, the culture plates were examined for bacterial growth, colony morphology, lactose fermentation, hemolysis, and other characteristic features. The bacterial isolates were identified by conventional microbiological methods based on Gram staining, colony morphology, and standard biochemical tests. Antimicrobial susceptibility testing was performed by the Kirby-Bauer disc diffusion method on Mueller-Hinton agar, and the results were interpreted according to the Clinical and Laboratory Standards Institute (CLSI) 2024 guidelines [8].

Definition of Multidrug Resistance

Multidrug resistance was defined as resistance to at least one antimicrobial agent in three or more antimicrobial classes [3]. Based on this definition, 92 MDR-GNB were included for final analysis.

Phenotypic detection of beta-lactamase enzymes among Gram-negative bacterial isolates

All MDR-GNB were screened for the phenotypic detection of beta-lactamase enzymes, including ESBL, MBL, and AmpC beta-lactamase. For ESBL detection, ceftazidime and ceftazidime-clavulanic acid discs were used. An increase of ≥5 mm in the zone diameter around the ceftazidime-clavulanic acid disc compared with the ceftazidime disc alone was considered positive for ESBL production [9]. MBL production was detected using imipenem and imipenem-EDTA discs. An increase of ≥7 mm in the inhibition zone diameter around the imipenem-EDTA disc compared with the imipenem disc alone was considered positive for MBL production [9]. AmpC beta-lactamase production was detected using cefoxitin and cefoxitin-cloxacillin discs. An increase of >4 mm in the zone diameter around the cefoxitin-cloxacillin disc compared with the cefoxitin disc alone was considered positive for AmpC beta-lactamase production [9,10].

CSE susceptibility testing

**Figure 1:**
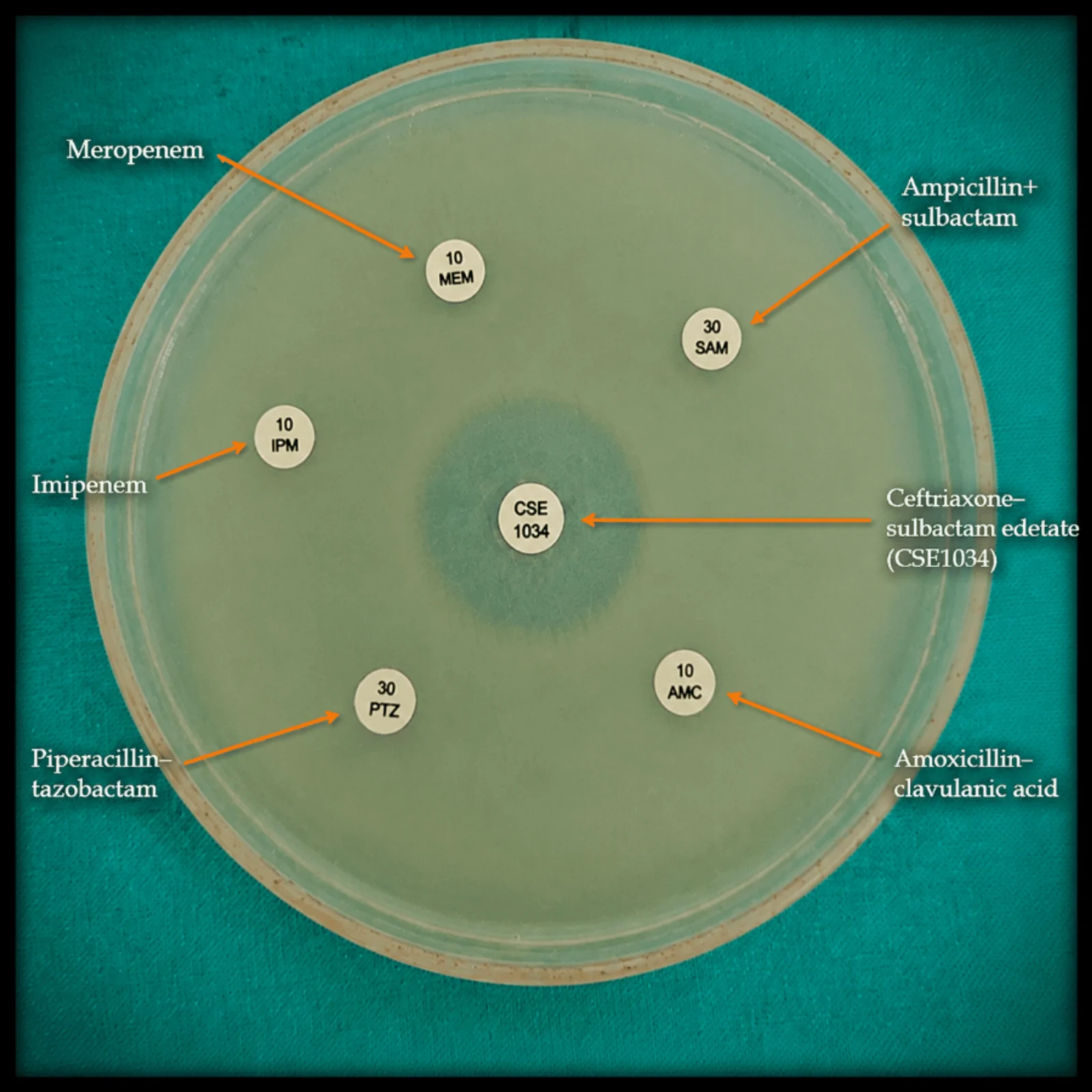
Ceftriaxone-sulbactam-EDTA (CSE) susceptibility testing by the Kirby-Bauer disc diffusion method. The figure shows the inhibition zone produced by the CSE disc against a representative multidrug-resistant (MDR) Gram-negative isolate. The measured inhibition-zone diameter was 25 mm, which was interpreted as susceptible according to the manufacturer's recommended criteria.

CSE susceptibility testing was performed for all MDR-GNB by the Kirby-Bauer disc diffusion method on Mueller-Hinton agar as shown in Figure 1. The bacterial inoculum was adjusted to 0.5 McFarland standard and lawn-cultured on Mueller-Hinton agar. The CSE disc was placed on the surface of Mueller-Hinton agar, and the plates were incubated aerobically at 37°C for 18-24 hours. After incubation, the diameter of the inhibition zone around the CSE disc was measured in millimeters. The results were interpreted according to the manufacturer-recommended criteria, where a zone diameter of ≥23 mm was considered susceptible and ≤22 mm was considered resistant [11,12].

## Results

In the present study, a total of 92 MDR-GNB were isolated from ICU-admitted patients with suspected infections. Among these isolates, 69 (75.00%) were from male patients and 23 (25.00%) were from female patients, indicating male predominance. The maximum number of isolates was reported in the 31-40 and 41-50 age groups, with 26 (28.26%) isolates in each group. In the 31-40 group, 18 (19.57%) were males, and 8 (8.70%) were females, while in the 41-50 group, 19 (20.65%) were males and 7 (7.61%) were females. The least number of isolates was observed in the 11-20 and 71-80 age groups, with 2 (2.17%) isolates each (Table 1).

**Table 1:** Age- and gender-wise distribution of multidrug-resistant Gram-negative bacilli (MDR-GNB) isolates from ICU patients. n: Number; %: Percentage; ICU: Intensive care unit; MDR-GNB: Multidrug-resistant Gram-negative bacilli.

Age group (year)	Male n (%)	Female n (%)	Total n (%)
11-20	1 (1.09)	1 (1.09)	2 (2.17)
21-30	12 (13.04)	6 (6.52)	18 (19.57)
31-40	18 (19.57)	8 (8.70)	26 (28.26)
41-50	19 (20.65)	7 (7.61)	26 (28.26)
51-60	8 (8.70)	3 (3.26)	11 (11.96)
61-70	9 (9.78)	5 (5.43)	14 (15.22)
71-80	2 (2.17)	0 (0.00)	2 (2.17)
Total	69 (75.00)	23 (25.00)	92 (100.00)

Among the 92 MDR-GNB isolates included in the study, urine was the most common clinical specimen, accounting for 39 (42.39%) isolates, followed by endotracheal tube secretions with 20 (21.74%) isolates. Sputum accounted for 11 (11.96%) isolates, while pus/wound swab samples contributed 9 (9.78%) isolates. Blood samples yielded eight (8.70%) isolates, and ascitic and peritoneal fluids accounted for five (5.43%) isolates. Thus, urine was the predominant source of MDR-GNB isolates among ICU patients, followed by respiratory specimens (Table 2).

**Table 2:** Specimen-wise distribution of MDR-GNB among clinical ICU isolates. n: Number; %: Percentage; MDR-GNB: Multidrug-resistant Gram-negative bacilli; ETT: Endotracheal tube secretion; ICU: Intensive care unit.

Sample type	Number (n)	Percentage %
Urine	39	42.39
ETT secretion	20	21.74
Sputum	11	11.96
Pus/Wound swab	9	9.78
Ascitic & peritoneal fluids	5	5.43
Blood	8	8.70
Total	92	100.00

**Figure 2:**
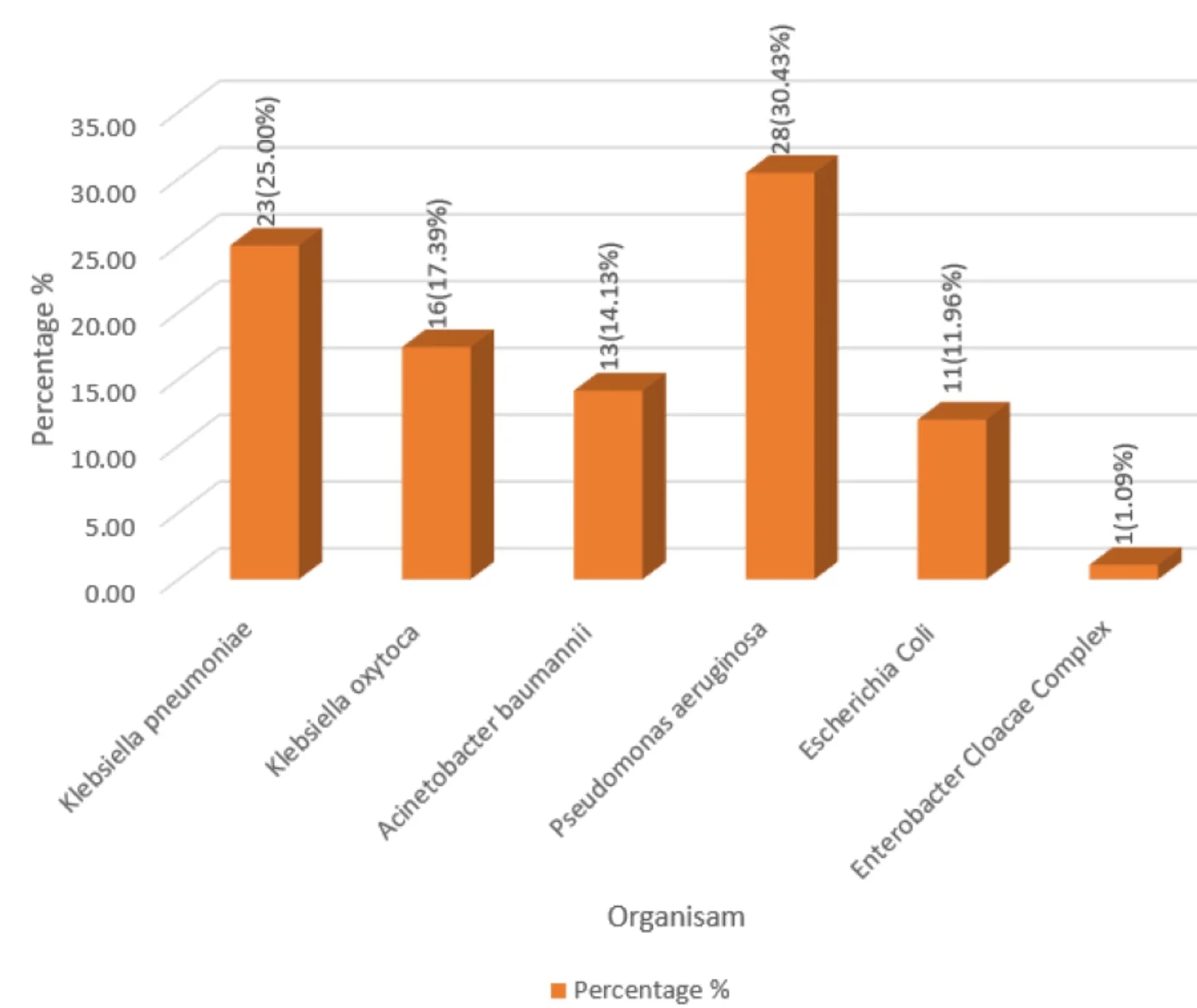
Organism-wise distribution of MDR-GNB among clinical ICU isolates. MDR-GNB: Multidrug-resistant Gram-negative bacilli; ICU: Intensive care unit.

In the organism-wise distribution of 92 MDR-GNB isolates,* P. aeruginosa *was the most commonly isolated organism, accounting for 28 (30.43%) isolates. This was followed by* K. pneumoniae *with 23 (25.00%) isolates and *Klebsiella oxytoca *with 16 (17.39%) isolates. *A. baumannii* accounted for 13 (14.13%) isolates, while *E. coli* contributed 11 (11.96%) isolates. *Enterobacter cloacae complex* was the least commonly isolated organism, with 1 (1.09%) isolate. Thus, *P. aeruginosa* was the predominant MDR-GNB isolate among ICU clinical specimens (Figure 2).

Among the 92 MDR-GNB isolates, 51 isolates were ESBL producers, 48 were MBL producers, and 38 were AmpC producers. Among ESBL-producing isolates, *P. aeruginosa* was the most common organism, accounting for 14 (27.45%) isolates, followed by *K. pneumoniae* with 11 (21.57%) isolates, *A. baumannii* with 10 (19.61%) isolates, *E. coli* with 9 (17.65%) isolates, *K. oxytoca* with 6 (11.76%) isolates, and *E. cloacae complex* with 1 (1.96%) isolate.

Among MBL-producing isolates, *K. pneumoniae* and *P. aeruginosa* were the predominant organisms, with 12 (25.00%) isolates each, followed by *K. oxytoca* with 10 (20.83%) isolates, *A. baumannii* with 7 (14.58%) isolates, *E. coli* with 6 (12.50%) isolates, and *E. cloacae complex* with 1 (2.08%) isolate.

Among AmpC-producing isolates, *P. aeruginosa* was the most frequent organism, accounting for 14 (36.84%) isolates, followed by *E. coli* with 9 (23.68%) isolates. *K. pneumoniae* and *A. baumannii* accounted for 7 (18.42%) isolates each, while *K. oxytoca* contributed 1 (2.63%) isolate. No AmpC production was observed in *E. cloacae complex *(Table 3).

**Table 3:** Distribution of ESBL, MBL, and AmpC-producing MDR-GNB ICU isolates. n: Number; %: Percentage; ESBL: Extended-spectrum beta-lactamase; MBL: Metallo-beta-lactamase; AmpC: AmpC beta-lactamase; MDR-GNB: Multidrug-resistant Gram-negative bacilli; ICU: Intensive care unit.

Organisms	ESBL producers n (%)	MBL producers n (%)	AmpC producers n (%)
K. oxytoca	6 (11.76)	10 (20.83)	1 (2.63)
K. pneumoniae	11 (21.57)	12 (25.00)	7 (18.42)
A. baumannii	10 (19.61)	7 (14.58)	7 (18.42)
P. aeruginosa	14 (27.45)	12 (25.00)	14 (36.84)
E. coli	9 (17.65)	6 (12.50)	9 (23.68)
E. cloacae complex	1 (1.96)	1 (2.08)	0 (0.00)
Total	51 (100.00)	48 (100.00)	38 (100.00)

**Figure 3:**
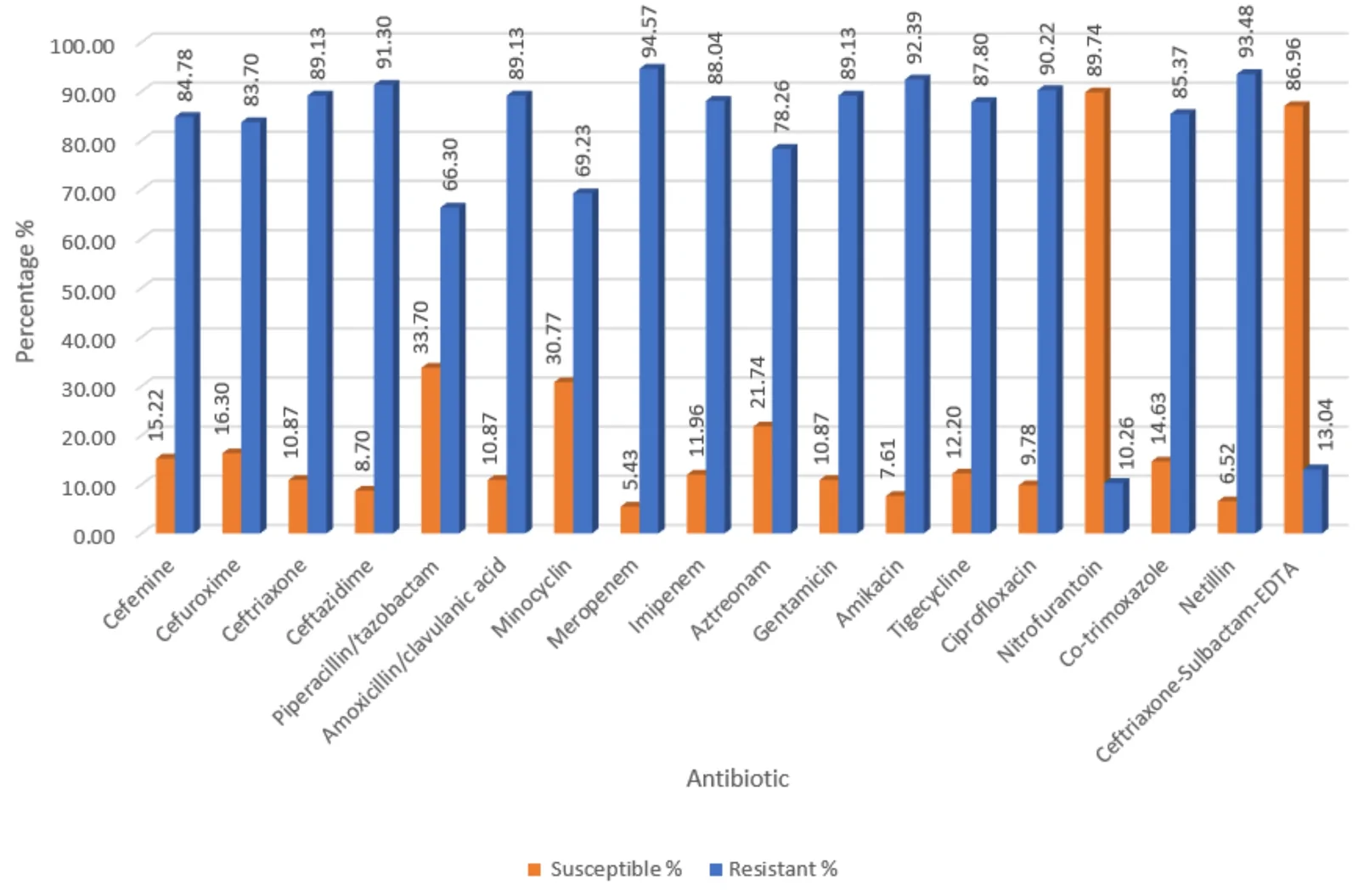
Comparison of CSE susceptibility with other antibiotics among MDR-GNB clinical ICU isolates. n: Number; %: Percentage; MDR-GNB: Multidrug-resistant Gram-negative bacilli; ICU: Intensive care unit.

Among the 92 MDR-GNB isolates, CSE demonstrated the highest *in vitro* susceptibility, with 80 (86.96%) isolates susceptible and 12 (13.04%) resistant. Nitrofurantoin susceptibility testing was performed only for urinary isolates (n = 39), among which 35 (89.74%) were susceptible and 4 (10.26%) were resistant. High levels of resistance were observed against meropenem, with 87 (94.57%) resistant isolates, followed by netilmicin 86 (93.48%), amikacin 85 (92.39%), ceftazidime 84 (91.30%), and ciprofloxacin 83 (90.22%). Resistance to ceftriaxone and amoxicillin/clavulanic acid was observed in 82 (89.13%) isolates each, while imipenem showed resistance in 81 (88.04%) isolates. Among the other antibiotics tested against the 92 MDR-GNB isolates, piperacillin/tazobactam showed comparatively higher susceptibility, with 31 (33.70%) susceptible isolates, followed by aztreonam with 20 (21.74%) susceptible isolates. Cefuroxime showed susceptibility in 15 (16.30%) isolates, while cefepime showed susceptibility in 14 (15.22%) isolates. Gentamicin showed susceptibility in 10 (10.87%) isolates. Minocycline was tested only against *A. baumannii *isolates (n = 13), of which 4 (30.77%) were susceptible and 9 (69.23%) were resistant. Tigecycline susceptibility testing was performed in 41 eligible MDR-GNB isolates, excluding *P. aeruginosa* and *A.* *baumannii*. Among these, five (12.20%) isolates were susceptible and 36 (87.80%) were resistant. Co-trimoxazole was not tested against *A. baumannii* or *P. aeruginosa* and was evaluated among 41 eligible isolates, of which 6 (14.63%) were susceptible and 35 (85.37%) were resistant. Overall, CSE demonstrated substantial *in vitro* activity against MDR-GNB, with susceptibility observed in 80 of 92 (86.96%) isolates, whereas most of the commonly tested antibiotics showed high levels of resistance (Figure 3).

## Discussion

MDR-GNBs represent a major challenge in ICUs. Critically ill patients are at increased risk of healthcare-associated infections due to prolonged hospital stays, use of invasive devices such as urinary catheters, endotracheal tubes (ETT), and central venous lines, and frequent exposure to broad-spectrum antibiotics. These factors create favorable conditions for the emergence and spread of resistant organisms [12,13]. The present study evaluated the distribution of MDR-GNB within the selected collection of 92 ICU MDR clinical isolates, identified the predominant bacterial pathogens, and assessed their distribution according to age and gender. In addition, antimicrobial susceptibility and resistance patterns were analyzed to determine the effectiveness of commonly used antibiotics. These findings provide useful local *in vitro* susceptibility data that may support antimicrobial stewardship and strengthen infection control practices in ICU settings.

In the present study, MDR-GNB isolates were most commonly observed in the 31-40 and 41-50 age groups, with 26 (28.26%) isolates each, followed by the 21-30 age group with 18 (19.57%) isolates. The lowest number of isolates was seen in the 11-20 and 71-80 age groups, with two (2.17%) isolates each. Gender-wise, male predominance was observed, with 69 (75.00%) isolates from male patients and 23 (25.00%) from female patients. Similar findings were reported by Maina et al. and Çelik et al., who also observed MDR Gram-negative bacterial infections among ICU patients and highlighted ICU-related risk factors such as prolonged hospitalization, invasive device use, and prior antibiotic exposure [14,15].

Specimen-wise distribution of MDR-GNB among clinical ICU isolates showed that urine was the most common specimen, accounting for 39 (42.39%) isolates, followed by ETT secretions with 20 (21.74%) isolates. Sputum contributed 11 (11.96%) isolates, while pus/wound swabs accounted for nine (9.78%) isolates. Blood samples showed eight (8.70%) isolates, and ascitic/peritoneal fluids accounted for five (5.43%) isolates. The predominance of urine and respiratory specimens may be due to frequent use of urinary catheters, endotracheal intubation, prolonged ICU stay, and exposure to broad-spectrum antibiotics. Similar findings were reported by Maina et al., who observed MDR Gram-negative bacterial infections among ICU patients and identified invasive device use and prolonged hospitalization as important risk factors [14]. Gong et al. also highlighted different infection profiles and antimicrobial resistance patterns among ICU patients, emphasizing the burden of resistant Gram-negative infections in critical care settings [16].

Organism-wise distribution of MDR-GNB among clinical ICU isolates showed that *P. aeruginosa* was the most commonly isolated organism, accounting for 28 (30.43%) isolates. This was followed by *K. pneumoniae *with 23 (25.00%) isolates and* K. oxytoca* with 16 (17.39%) isolates. *A. baumannii *accounted for 13 (14.13%) isolates, while *E. coli *contributed 11 (11.96%) isolates. *E. cloacae complex* was the least commonly isolated organism, with 1 (1.09%) isolate. The predominance of* P. aeruginosa* and *Klebsiella species* may be due to their frequent association with ICU-acquired infections, invasive device use, biofilm formation, prolonged hospitalization, and exposure to broad-spectrum antibiotics. Similar findings were reported by Bassetti et al., who highlighted the clinical importance of MDR Gram-negative pathogens such as* P. aeruginosa, A. baumannii, *and Enterobacteriaceae in hospital settings [17]. Gong et al. also reported variations in infection profiles and antimicrobial resistance patterns among ICU patients, emphasizing the burden of MDR Gram-negative organisms in critical care units [16]. Girotra et al. further reported the activity of CSE against MDR *A. baumannii, P. aeruginosa,* and Enterobacteriaceae isolates, which are considered important WHO critical priority pathogens [18].

In the present study, phenotypic distribution of beta-lactamase-producing MDR-GNB isolates showed that ESBL production was observed in 51 isolates, MBL production in 48 isolates, and AmpC production in 38 isolates. Among ESBL producers, *P. aeruginosa *was the most common organism, accounting for 14 (27.45%) isolates, followed by *K. pneumoniae* with 11 (21.57%) isolates and *A. baumannii *with 10 (19.61%) isolates. Among MBL producers, *K. pneumoniae* and *P. aeruginosa* were the predominant organisms, with 12 (25.00%) isolates each, followed by *K. oxytoca* with 10 (20.83%) isolates. AmpC production was highest among* P. aeruginosa,* with 14 (36.84%) isolates, followed by* E. coli *with 9 (23.68%) isolates, and *K. pneumoniae* and *A. baumannii *with 7 (18.42%) isolates each.

The high occurrence of ESBL, MBL, and AmpC producers indicates the significant burden of beta-lactamase-mediated resistance among ICU MDR-GNB isolates. This may be due to prolonged ICU stay, frequent use of broad-spectrum antibiotics, invasive procedures, and horizontal spread of resistance genes. Similar findings were reported by Fatima et al., who studied resistant Enterobacteriaceae producing ESBLs and MBLs. Chaudhary and Payasi also highlighted the role of ESBL-producing organisms and biofilm formation in antibiotic resistance. These findings emphasize the need for routine detection of beta-lactamase producers, strict infection control measures, and rational antibiotic use in ICU settings [9,11].

Comparison of CSE susceptibility with other antibiotics showed that CSE had the highest susceptibility, with 80 (86.96%) isolates susceptible and 12 (13.04%) resistant. Piperacillin/tazobactam showed susceptibility in 31 (33.70%) isolates, followed by aztreonam in 20 (21.74%). High resistance was observed to meropenem (94.57%), netilmicin (93.48%), amikacin (92.39%), ceftazidime (91.30%), and ciprofloxacin (90.22%). Nitrofurantoin showed good activity among urinary isolates, with 35 (89.74%) susceptible. Minocycline showed susceptibility in four (30.77%) *A. baumannii* isolates. Co-trimoxazole showed susceptibility in six (14.63%) of 41 eligible isolates, while tigecycline showed susceptibility in five (12.20%) of 41 eligible isolates.

The higher susceptibility observed with CSE indicates promising *in vitro* activity against MDR-GNB isolates. Similar findings were reported by Paul et al., who observed good *in vitro* susceptibility of MDR Gram-negative bacterial isolates to CSE [12]. However, these findings are based on *in vitro* susceptibility testing and should not be interpreted as evidence of clinical efficacy or as a direct therapeutic recommendation. Susceptibility-guided antimicrobial therapy, routine resistance screening, strict infection control measures, and antimicrobial stewardship remain essential for the management of MDR-GNB infections in ICU settings.

Limitations

This study was conducted in a single tertiary care hospital, so the findings may not represent MDR-GNB patterns in other healthcare settings. The sample size was limited to ICU clinical isolates, and molecular characterization of resistance genes was not performed. Therefore, further multicentric studies with larger sample sizes and molecular confirmation are needed to better understand resistance mechanisms and guide effective treatment strategies.

## Conclusions

MDR-GNB were frequently identified among the selected Gram-negative isolates recovered from ICU clinical specimens, with the highest proportion of isolates observed in the 31-40 and 41-50 age groups and a male predominance. Urine was the most common specimen source, followed by endotracheal tube secretions and sputum. *P. aeruginosa* and *K. pneumoniae* were the predominant organisms. High resistance to commonly used antibiotics and the presence of ESBL, MBL, and AmpC producers indicate a serious antimicrobial resistance burden in ICU settings. CSE demonstrated good *in vitro* activity against the selected MDR-GNB isolates based on the applied susceptibility criteria. However, these findings represent laboratory-based *in vitro* activity and should not be interpreted as evidence of clinical efficacy or as a therapeutic recommendation.
